# Maternal levels of acute phase proteins in early pregnancy and risk of autism spectrum disorders in offspring

**DOI:** 10.1038/s41398-022-01907-z

**Published:** 2022-04-07

**Authors:** Martin Brynge, Renee Gardner, Hugo Sjöqvist, Håkan Karlsson, Christina Dalman

**Affiliations:** 1 Department of Global Public Health, Karolinska Institutet, Stockholm, 17177 Sweden; 2 Department of Neuroscience, Karolinska Institutet, Stockholm, 17177 Sweden; 3 Centre for Epidemiology and Community Medicine, Region Stockholm, Stockholm, 17129 Sweden

**Keywords:** Predictive markers, Molecular neuroscience

## Abstract

Previous research supports a contribution of early-life immune disturbances in the etiology of autism spectrum disorders (ASD). Biomarker studies of the maternal innate (non-adaptive) immune status related to ASD risk have focused on one of the acute phase proteins (APP), C-reactive protein (CRP), with conflicting results. We evaluated levels of eight different APP in first-trimester maternal serum samples, from 318 mothers to ASD cases and 429 mothers to ASD-unaffected controls, nested within the register-based Stockholm Youth Cohort. While no overall associations between high levels of APP and ASD were observed, associations varied across diagnostic sub-groups based on co-occurring conditions. Maternal levels of CRP in the lowest compared to the middle tertile were associated with increased risk of ASD without ID or ADHD in offspring (OR = 1.92, 95% CI 1.08–3.42). Further, levels of maternal ferritin in the lowest (OR = 1.78, 95% CI 1.18–2.69) and highest (OR = 1.64, 95% CI 1.11–2.43) tertiles were associated with increased risk of any ASD diagnosis in offspring, with stronger associations still between the lowest (OR = 3.81, 95% CI 1.91–7.58) and highest (OR = 3.36, 95% CI 1.73–6.53) tertiles of ferritin and risk of ASD with ID. The biological interpretation of lower CRP levels among mothers to ASD cases is not clear but might be related to the function of the maternal innate immune system. The finding of aberrant levels of ferritin conferring risk of ASD-phenotypes indicates a plausibly important role of iron during neurodevelopment.

## Introduction

The etiology of autism spectrum disorders (ASD) is complex, with plausible contributions from both genetic variation and early environmental exposures [1]. ASD often co-occurs with other developmental disorders such as intellectual disability (ID) and attention deficit/hyperactivity disorder (ADHD) [2, 3] defining diagnostic sub-groups with potentially different etiological pathways in terms of genetic and environmental contributions [4, 5].

Evidence that immune-related proteins play pleiotropic roles during neurodevelopment and observations of immune anomalies among ASD-affected individuals [6, 7] have led to the hypothesis that immune dysregulation during early life increases risk of ASD [7–9]. Experimental animal models of maternal immune activation report functional and morphological changes in the brain of exposed offspring as well as autism-like phenotypes in the offspring, with decreased socialization and restricted patterns of behavior [10, 11]. However, the validity of such animal models for ASD remains to be established [12]. Nevertheless, observational studies suggest that events associated with systemic inflammation, such as maternal autoimmune disease, obesity, and infections during pregnancy, are linked to ASD [11, 13–17]. The role of inflammation per se in the mechanisms underlying these associations is uncertain, and the observed associations may also partly be explained by genetic confounding [18–20].

To understand if the maternal immune activation hypothesis is relevant for ASD, numerous studies have explored maternal immune-related biomarkers from blood samples collected during pregnancy. These studies have mostly focused on cytokines, with inconsistent results [21–23]. Cytokines are powerful regulators of the immune system that also play roles in CNS development, but quantitation is technically challenging due to generally low baseline levels with transient secretion patterns and short half-lives. Acute phase proteins (APP) are regulated by cytokines and are involved in the initial non-adaptive (innate) immune response [24], although several APP exhibit additional functions, not apparently related to immune function [25–27]. They generally have longer half-lives compared to cytokines and baseline concentrations in the measurable range. The few previous studies on gestational APP and risk of ASD, measured only one of the APP, C-reactive protein (CRP) [28–31], with inconsistent results.

In the current study, we measured eight different APP in sera collected in the first trimester of pregnancy and estimated the risk of ASD in the offspring associated with the individual APP, using a large, well-characterized population-based cohort.

## Materials and methods

### Study population

The present study employed a case-control study design nested within the population-based register linkage Stockholm Youth Cohort [32]. The source population consisted of children born 1996–2000 (*n* = 98,597) (Supplementary Fig. 1). We sampled all possible cases of ASD and a random selection of the source population to serve as controls in this nested case-control study. In Stockholm County, maternal serum samples obtained during the national screening program of pregnant women have been stored frozen since 1998. We retrieved 0.2 ml serum from all available maternal sera samples, for ASD cases (*n* = 430) and mothers to control individuals (*n* = 549), from those individuals who also had a neonatal dried blood spot (NDBS) as detailed previously [33]. Samples were obtained at median gestational week 10.6 (interquartile range: 9.3–12.5 weeks) representing a period near the end of the first trimester.

For the analysis in this study, we included only those samples drawn in the first trimester (≤13 weeks), since maternal serum proteins may vary by gestational age due to maternal blood volume expansion and altered metabolism [34, 35]. In line with previous findings, we observed a relationship between CRP and gestational week at sampling (Supplementary Figure 2A) [28], among all individuals from whom a serum sample was collected, further justifying the restriction of samples to a specific interval of pregnancy. We also observed time-dependent patterns for several of the other APP, including ferritin (FER) (Supplementary Fig. 2B).

Our final study sample consisted of 318 mothers to ASD cases and 429 mothers to control individuals. Mothers to individuals in the final study sample were generally similar to the source population, though they tended to be older, have higher educational level, be of higher socioeconomic status, and were less likely to be born in Asia or Africa (Supplementary Table 1). We calculated that we would be able to detect an odds ratio of 1.58, after dividing the sample into tertiles based on the distribution of the measured APP (see analytical strategy below), at 80% power given the number of cases included.

Ethical approval was obtained by the Stockholm regional review board (DNR 2011/695-31/2, amendment 2012/706-32). Individual consent was not required for this anonymized register-based study.

### Case ascertainment

The case-finding procedure has been described previously [32, 33]. We considered any ASD diagnosis as an outcome (regardless of comorbidities) and also stratified the outcomes as ASD only (without comorbid ID or ADHD), ASD with ID, and ASD with ADHD. Individuals with both comorbid ID and ADHD were included in the ASD with ID group.

### Laboratory analysis

Serum aliquots were stored at −80 °C until analysis. After thawing on ice, samples were diluted 1:10 000 for analysis of α-2 macroglobulin (A2M), haptoglobin (HAP), CRP and serum amyloid P (SAP), or 1:100 for analysis of FER, fibrinogen (FIB), procalcitonin (PCT), serum amyloid A (SAA) and tissue plasminogen activator (tPA). Samples were assigned randomly to 96-well assay plates for analysis using commercially available premixed, multiplex panels (Bio-Rad, catalog number 171A4S07M) and the Bio-Plex 200 System (Bio-Rad, Hercules, CA, USA).

The average inter- and intra-assay coefficients of variation for manufacturer-provided control samples over the 13 assay plates were 18.6% and 8.6%, respectively (Supplementary Table 2). Values below the lower limit of quantitation (LLOQ) were assigned a value of LLOQ/√2. Values above the upper limit of quantitation (ULOQ) were assigned a value of ULOQ × 1.1. HAP had a large proportion of imputed values (75.77%), with the majority above the ULOQ, and was therefore excluded from the final analysis.

### Covariates

Covariates considered as potential confounders were chosen on the basis of previously described relationships with ASD or with a plausible relationship with APP: maternal age, psychiatric history, BMI, region of birth, education, smoking at first antenatal visit; fetal sex; birth order; family income; and gestational week and season at serum sampling. Covariates were extracted from the Medical Birth and National Patient Registers. Sociodemographic data were extracted from the Integrated Database for Labor Market Research.

### Statistical analysis

The concentrations of APP were log-transformed to normalize the distributions (Supplementary Fig. 3A). To reduce the influence of assay plate-to-plate variation, we calculated standardized plate-based z-scores by subtracting the plate-specific mean concentration from each measurement and dividing by the plate-specific standard deviation (Supplementary Fig. 3B). The standardized scores were then categorized into tertiles based on the distribution among controls (Supplementary Fig. 3C). All statistical tests performed in the study were two-sided.

Covariates were tested for association with the z-scores of each APP among mothers to cases and controls separately. We conducted linear regression analyses with the covariates as the independent variable and each APP as outcome variables, followed by a joint Wald test to examine if the categories of the covariates were associated with different mean levels of APP. Covariates were included in the adjusted models if even weakly associated (*p* < 0.2) with at least one of the APP among mothers to the control population and with any of the outcomes. The final adjusted model included sex; birth order; family income quintile; maternal BMI, psychiatric history, region of origin, and age.

In the categorical analyses, we used logistic regression models to estimate the odds of ASD associated with the different APP, using the middle tertile as the referent and followed by a Wald test for the association between each APP and ASD. For continuous analyses, we used restricted cubic spline models with three knots and a *z*-score = 0 as the referent. To examine the overall association between each APP and ASD, we tested if all spline terms jointly were equal to zero using the Wald test. We investigated evidence for potential non-linearity by testing if all spline terms that would indicate a change in the direction of the relationship were equal to zero.

We conducted sensitivity analyses by restricting the cohort to the sample of Nordic-born mothers, as maternal region of origin was associated with some APP and also with the likelihood of a child receiving an ASD with ID diagnosis. The non-Nordic group (*n* = 148) was too small to evaluate on its own. In a separate sensitivity analysis, we adjusted additionally for annual quarter (season) at serum sampling and gestational week at serum sampling, factors that were not associated with ASD, but plausibly with APP levels.

All statistical analyses were conducted using Stata (v. 14.1) with external package xbrcspline [36]. Code is available from the corresponding author upon request.

## Results

### Association of covariates with case status

As expected, ASD cases were more likely to be male and firstborn (Supplementary Table 3). Mothers to ASD cases were more likely to have a history of psychiatric illness, have lower family income levels, and were less likely to have a normal BMI, compared with mothers to unaffected controls. Mothers born in Africa and Asia were overrepresented among children with ASD and comorbid ID.

### Association of APP with each other and with covariates

Among mothers to unaffected controls, 14 of the 28 possible pairwise combinations of maternal APP (Supplementary Fig. 4) were positively correlated at a significance level *p* < 0.05, with the strongest correlations observed between CRP and A2M; CRP and SAP; A2M and SAP; and tPA and PCT.

**Fig. 1:**
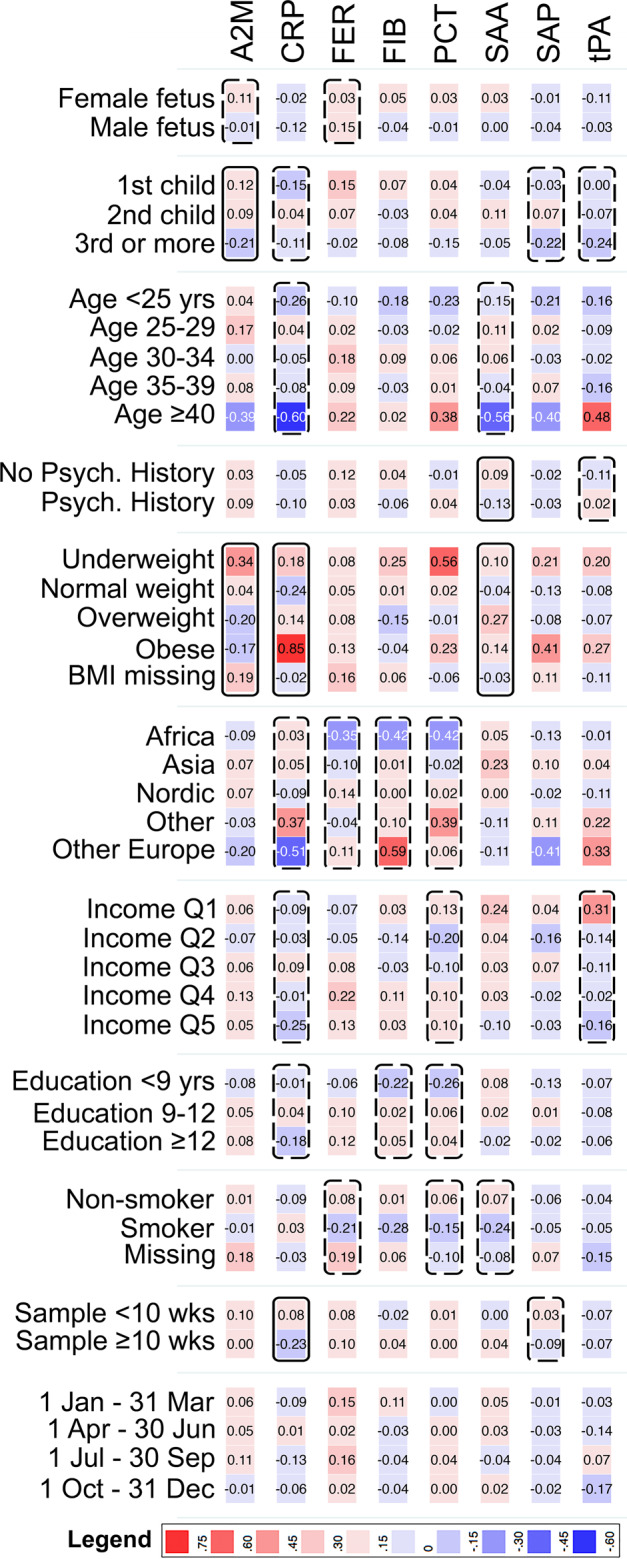
Heat map showing the mean maternal APP z-score by categories of the covariates, among mothers to 429 unaffected individuals in the cohort. Solid boxes indicate that the APP is associated with the covariate at *p* < 0.05. Dashed boxes indicate that the APP is associated with the covariate at *p* < 0.20. Abbreviations: A2M α-2 macroglobulin, CRP C-reactive protein, FER ferritin, FIB fibrinogen, PCT procalcitonin, SAA serum amyloid A, SAP serum amyloid P, tPA tissue plasminogen activator, Psych Psychiatric, BMI body mass index, Income Q income quintile.

Associations (*p* < 0.2) with at least one APP were seen for all covariates except serum sampling quarter in mothers to unaffected individuals (Fig. 1, Supplementary Table 4). Maternal BMI was among the strongest predictors of APP levels. Similarly, among mothers to ASD-affected individuals, all covariates were associated (*p* < 0.2) with at least one APP, except sex and maternal age (Supplementary Fig. 5, Supplementary Table 5).

### Association of APP with odds of ASD

Compared with mothers to ASD-unaffected controls, there were no significant differences in median levels of APP in mothers to ASD-affected individuals, though there was a reduction (*p* < 0.05) in CRP among mothers to offspring with ASD only (Supplementary Table 3, Supplementary Fig. 6).

In the unadjusted regression analysis of APP tertiles, we observed a trend toward a u-shaped association (*p* = 0.06) between FER and odds of any ASD (Supplementary Fig. 7), with a similar u-shaped pattern present for the stratified outcome ASD with ID (*p* < 0.01) but absent for the other stratified outcomes. The lowest tertile of CRP was associated with increased odds of ASD only (*p* < 0.01).

**Fig. 2:**
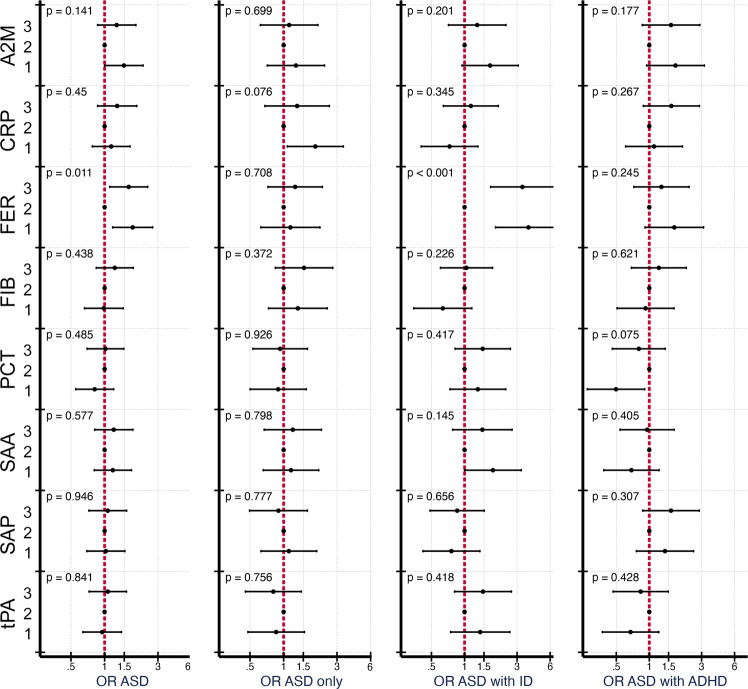
The relationship between maternal APP measured prenatally and odds of ASD, stratified by co-occurrence of ID and ADHD, when comparing mothers of 318 ASD cases to mothers of 429 unaffected individuals selected from the cohort. Tertiles of each APP were created using the distribution of z-scores among unaffected individuals to set the cut-offs, and the middle quintile was used as the referent category. Models were adjusted for sex, birth order, maternal BMI, maternal psychiatric history, maternal region of origin, maternal age and family income quintile. Error bars represent the 95% confidence interval for the fully adjusted model. *P* values are shown for a Wald test with a null hypothesis that all APP categorical terms were jointly equal to zero, as a test of whether each APP was generally associated with the outcome. Abbreviations: A2M α-2 macroglobulin, CRP C-reactive protein, FER ferritin, FIB fibrinogen, PCT procalcitonin, SAA serum amyloid A, SAP serum amyloid P, tPA tissue plasminogen activator.

In fully adjusted models, the association between FER and any ASD was strengthened with increased odds of ASD in the lowest (OR = 1.78, 95% CI 1.18–2.69) and highest (1.64, 95% CI 1.11–2.43) tertiles compared to the middle tertile (Fig. 2). The association between FER and odds of ASD with ID was also strengthened compared to the unadjusted analysis, with increased odds of ASD with ID for the lowest (3.81, 95% CI 1.91–7.58) and highest (3.36, 95% CI 1.73–6.53) tertiles (Wald test for overall association between FER and ASD with ID: *p* = 0.0003). The association between CRP and odds of ASD only was attenuated, although with increased odds for the lowest tertile compared to the middle tertile (1.92, 95% CI 1.08–3.42: Wald test for overall association between CRP and ASD only: *p* = 0.076). We also observed an association between the lowest tertile of PCT and decreased odds of ASD with ADHD (0.49, 95% CI 0.27-0.91; Wald test for overall association between PCT and ASD with ADHD: *p* = 0.075). When considering the multiple comparisons that were made, only the overall association between FER and ASD with ID fell below the Bonferroni adjusted p-value of 0.0008 (based on 64 statistical comparisons in spline and tertile models).

**Fig. 3:**
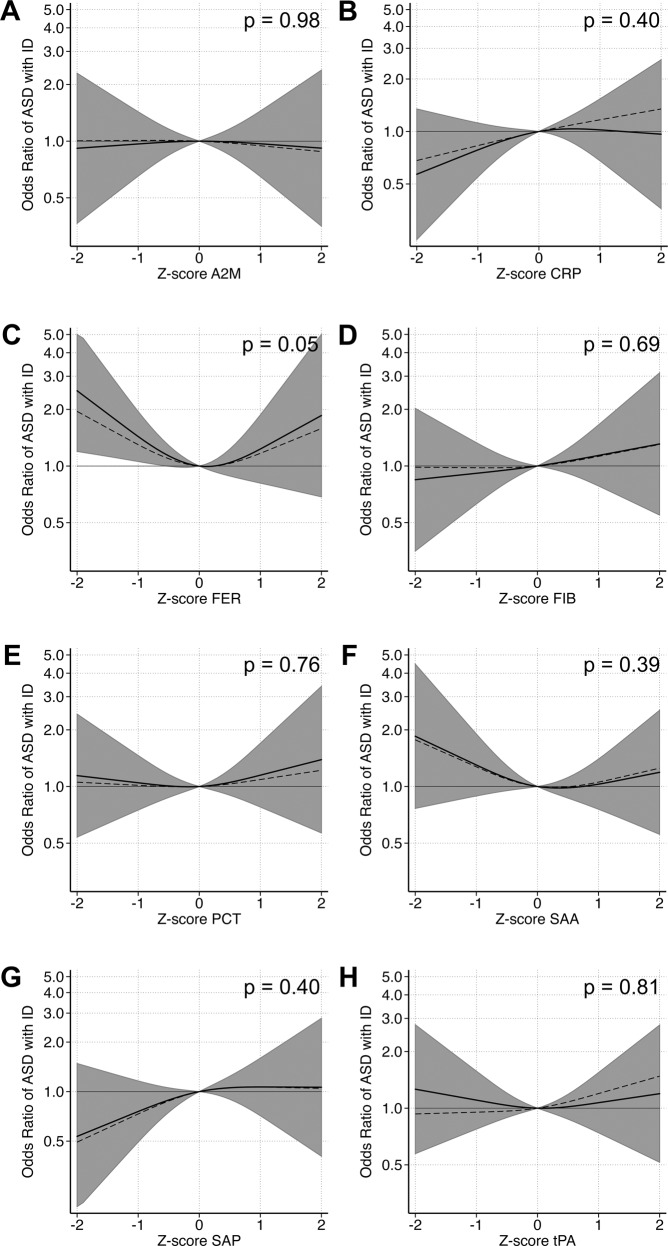
The relationship between maternal APP measured prenatally and odds of ASD with ID when comparing mothers of 101 individuals affected by ASD with ID to mothers of 429 unaffected individuals selected from the cohort. Each panel displays the odds of ASD with ID according to APP z-score, flexibly fit using restricted cubic spline models with three knots and a z-score = 0 as the referent. The dashed line represents the unadjusted estimate of the relationship between each APP and odds of ASD with ID. The solid line represents the fully adjusted model, adjusted for sex, birth order, maternal BMI, maternal psychiatric history, maternal region of origin, maternal age and family income quintile. The gray bands represent the 95% confidence interval for the fully adjusted model. *P* values are shown for a Wald test with a null hypothesis that all APP spline terms were jointly equal to zero, as a test of whether each APP was generally associated with the outcome. Abbreviations: A2M α-2 macroglobulin, CRP C-reactive protein, FER ferritin, FIB fibrinogen, PCT procalcitonin, SAA serum amyloid A, SAP serum amyloid P, tPA tissue plasminogen activator.

**Fig. 4:**
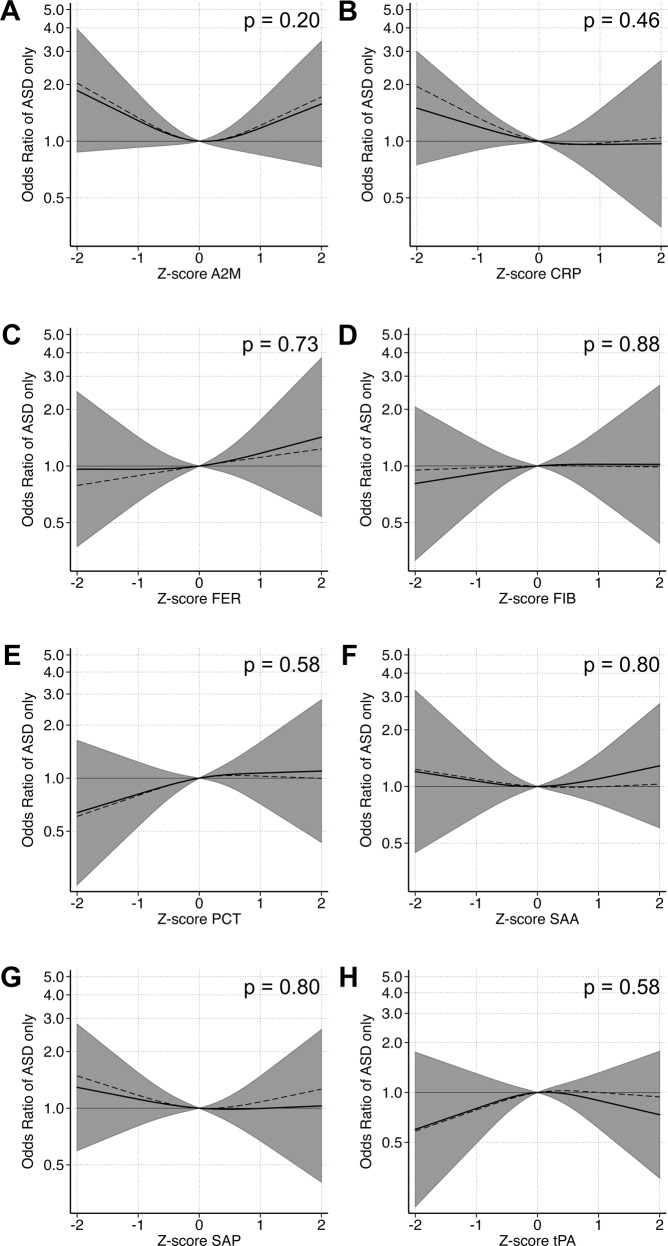
The relationship between maternal APP and odds of ASD without co-occurring ID or ADHD when comparing mothers of 100 individuals affected by ASD without co-occurring ID or ADHD to mothers of 429 unaffected individuals selected from the cohort. Each panel displays the odds of ASD without co-occurring ID or ADHD according to APP z-score, flexibly fit using restricted cubic spline models with three knots and a z-score = 0 as the referent. The dashed line represents the unadjusted estimate of the relationship between each APP and odds of ASD without co-occurring ID or ADHD. The solid line represents the fully adjusted model, adjusted for sex, birth order, maternal BMI, maternal psychiatric history, maternal region of origin, maternal age and family income quintile. The gray bands represent the 95% confidence interval for the fully adjusted model. *P* values are shown for a Wald test with a null hypothesis that all APP spline terms were jointly equal to zero, as a test of whether each APP was generally associated with the outcome. Abbreviations: A2M α-2 macroglobulin, CRP C-reactive protein, FER ferritin, FIB fibrinogen, PCT procalcitonin, SAA serum amyloid A, SAP serum amyloid P, and tPA tissue plasminogen activator.

**Fig. 5:**
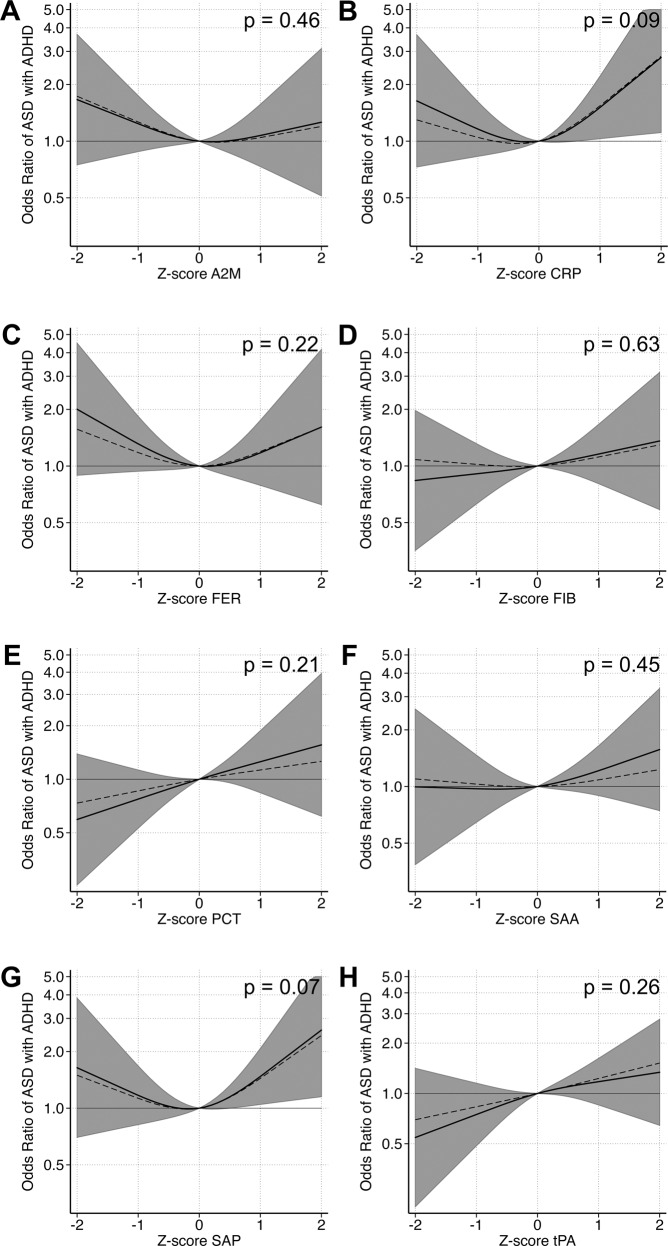
The relationship between maternal APP and odds of ASD with co-occurring ADHD when comparing mothers of 117 individuals affected by ASD with co-occurring ADHD to mothers of 429 unaffected individuals selected from the cohort. Each panel displays the odds of ASD with co-occurring ADHD according to APP z-score, flexibly fit using restricted cubic spline models with three knots and a z-score = 0 as the referent. The dashed line represents the unadjusted estimate of the relationship between each APP and odds of ASD with co-occurring ADHD. The solid line represents the fully adjusted model, adjusted for sex, birth order, maternal BMI, maternal psychiatric history, maternal region of origin, maternal age and family income quintile. The gray bands represent the 95% confidence interval for the fully adjusted model. *P* values are shown for a Wald test with a null hypothesis that all APP spline terms were jointly equal to zero, as a test of whether each APP was generally associated with the outcome. Abbreviations: A2M α-2 macroglobulin, CRP C-reactive protein, FER ferritin, FIB fibrinogen, PCT procalcitonin, SAA serum amyloid A, SAP serum amyloid P, tPA tissue plasminogen activator.

In adjusted cubic spline models, we observed evidence for non-linear relationships between z-scores of maternal CRP, FER, and SAP and the outcomes (Supplementary Table 6). As in the categorical models, we observed a u-shaped relationship between FER and any ASD (*p* = 0.13) as well as ASD with ID (*p* = 0.048; Supplementary Fig. 8, Fig. 3). The overall pattern of association between CRP and odds of ASD only, with higher odds at lower concentrations, was similar to the categorical analysis, although it did not reach statistical significance (*p* = 0.46) (Fig. 4). A u-shaped pattern of association was observed between maternal CRP (*p* = 0.085) and SAP (*p* = 0.067) and odds of ASD with ADHD (Fig. 5), with the strongest associations above the mean.

### Sensitivity analyses

The estimates observed in the sensitivity analyses, either restricting to Nordic-born mothers or considering factors that might influence APP levels, were similar to those observed in the main analyses (Supplementary Figs. 9 and 10).

## Discussion

In this population-based cohort of pregnant women, we measured a range of immune markers to assess the possible link between innate immune activation in early pregnancy and subsequent ASD in offspring. Overall, we found no strong evidence for elevated APP being associated with increased odds of ASD, regardless of comorbidities. However, we did observe that low levels of CRP were associated with increased odds of ASD without ID or ADHD, with some analyses suggesting that higher levels of CRP and SAP are associated with higher risk of ASD with ADHD. We also found that both low and high levels of FER were associated with increased odds of ASD, particularly ASD with ID.

### Comparison with previous studies

There are four previous studies investigating maternal APP and risk of ASD in offspring, all measuring the acute phase reactant CRP.

In a study by Brown et al. [28], the highest quintile of gestational CRP in first and early second trimester was associated with increased odds for autism, and a positive association was apparent when CRP was treated as a continuous variable. The prevalence of ASD in that study (1132 cases of childhood autism in a birth cohort of 1.2 million, or 0.094%) stands in contrast to the present study, where we included all ICD and DSM-IV autism diagnoses, with a joint prevalence of 1.52%. Furthermore, we were able to control for a larger number of health- and sociodemographic covariates, such as BMI and socioeconomic status, which may also contribute to the discrepancy in the results.

In a recent study by Egorova et al., 62 biomarkers including CRP were measured in serum samples (gestational week 14) of 100 mothers to ASD cases and 100 unaffected controls [31]. No significant associations with CRP were found, though this smaller study had lower power to detect differences. Also, CRP was treated as a linear continuous variable, in contrast to the present study where we allowed for non-linear relationships.

Koks et al. reported an association between maternal CRP (measured before 18 weeks of gestation) and autistic traits in offspring at age 6, as measured by the continuous Social Responsiveness Scale in the Generation R cohort study [30]. The association was completely attenuated after controlling for maternal health-related factors and socioeconomic covariates, illustrating the importance of controlling for such parameters.

In the Californian Early Markers for Autism (EMA) case-control study, the third and fourth quartiles of CRP (at 15–19 weeks of gestation) compared to the lowest quartile were associated with a decreased risk of ASD [29], which is in general agreement with our current findings. However, we also observed an overall tendency toward a u-shaped relationship between CRP and odds of ASD, although the specific pattern varied with the presence of comorbidities. Although the EMA study used a similar, broad definition of the ASD phenotype, there was no stratification of the ASD group based on co-occurring conditions. To the best of our knowledge, no study to date has investigated the relationship between maternal APP and ASD with comorbid ADHD specifically. In a study by the same research group as the study by Brown et al. [28], using the same birth cohort, maternal CRP in the first and second trimesters was not associated with offspring risk of ADHD [37].

### Interpretation and potential mechanisms

Baseline concentrations of APP are influenced by both genetic and environmental factors. Previous studies have reported heritability estimates in adults for FIB (27–51%), tPA (23–27%), and CRP (10–65%), with estimates from a longitudinal twin study at around 50% for CRP, with stability over time [38–40].

We observed evidence of moderate to high pairwise correlations between several of the APP, possibly reflecting shared regulation. However, many of the correlations were weak or non-existent, implying partially independent modes of regulation or biological function, in line with previous knowledge of the APP [24]. In general, lower correlations were observed among APPs in maternal serum compared to those observed in neonatal samples [33]. In accordance with previous reports [41], we observed a strong correlation between maternal BMI and CRP, with overweight and obesity being associated with elevated levels.

Several immune proteins have dual roles, including a role as modulators of neurodevelopment. Animal models and observational studies suggest a harmful effect of maternal infections during pregnancy on the developing fetal brain, not only of pathogenic (teratogenic) microorganisms themselves, but also due to maternal inflammatory mediators crossing the fetal-placental barrier [10, 12]. This is often referred to as the maternal immune activation hypothesis of ASD. Based on the eight APP studied here, we found no convincing general trend of maternal immune activation early in ASD pregnancies. Overall, we observed a tendency toward a u-shaped association between CRP and ASD, although the patterns varied across the stratified outcomes. While elevated levels of CRP and SAP were associated with ASD with co-occurring ADHD, low levels of CRP were associated with increased odds of ASD without ID or ADHD. We also observed a borderline significant association between increased odds of ASD with ID and the lowest tertile of SAA, an APP with secretion kinetics resembling those of CRP [42].

Both CRP and SAP are members of the pentraxin protein family, synthesized by hepatocytes as a response to proinflammatory cytokines, particularly interleukin-6, and to a lesser degree interleukin-1 and tumor necrosis factor-α [43, 44]. Their exact function is not known, but they are both evolutionarily conserved proteins that bind common molecular patterns on the surface of pathogens and induce complement activation [40, 44].

Evidence of an association between low levels of maternal CRP and increased odds of ASD are now apparent in two large case-control/cohort studies of similar design [29]. In schizophrenia, another complex neuropsychiatric disorder sharing common genetic variation, and brain transcriptional dysregulation with autism [45, 46], genetic loci associated with high circulating levels of CRP have been found protective in Mendelian randomization studies [47]. This is in line with an observational study of several neonatal APP and risk of non-affective psychotic disorders in adulthood [48]. In our case-cohort study of neonatal APP measured in dried blood spots, we observed a u-shaped association between CRP and odds of ASD [33]. In the present study, a similar u-shaped tendency was observed for CRP when considering CRP as a continuous predictor, with an association between high levels of CRP and ASD with ADHD in particular, indicating potential differences in etiological pathways according to the presence of comorbid conditions.

It is not clear how low levels of maternal CRP in first trimester might influence the risk of ASD in offspring. Considering the opsonizing and complement activating functions of CRP, low levels might represent a suboptimal innate immune effector function. Further, low levels of CRP in the first trimester do not exclude maternal or fetal immune activation later in pregnancy, and the low concentrations early in pregnancy might influence risk of serious infections later on [33]. The exact cause of the observed elevations of CRP and SAP among mothers to individuals with ASD with co-occurring ADHD is not known, but they might be caused by either genetically determined elevated levels, or in response to some environmental factor, e.g. infection.

Serum FER is a sensitive marker of total body iron not bound to hemoglobin [49]. There is increasing evidence concerning the importance of iron metabolism for early neurodevelopment [50]. Apart from having a role in oxygen transport, iron functions as a cofactor in cytochrome reactions generating ATP and in enzymes involved in neuronal myelinization [51–53]. In an observational study, Schmidt et al. reported an association between self-reported low maternal iron intake prior to and during pregnancy and risk of autism in the offspring [54]. Maternal iron deficiency is the most common cause of anemia, and the latter is associated with several adverse birth-related and behavioral outcomes in the offspring, including small for gestational age, ASD, ADHD, and ID [16, 55, 56]. Low FER levels in neonates have been associated with adverse neurocognitive and behavioral outcomes [57–59]. In our previous case-cohort study of neonatal APP, we observed an increased risk of ASD with lower levels of FER if mothers had anemia during gestation [33]. In a separate discordant sibling comparison, levels of FER below the mean were strongly associated with increased odds of ASD.

In our cohort, maternal region of origin was associated with FER, with reduced levels in mothers from Africa or Asia (Fig. 1, Supplementary Fig. 5). The reason for this is not clear, but it might potentially be related to socioeconomic and/or nutritional factors. This finding is in line with our previously reported observation that the proportion of anemic mothers is markedly higher among immigrants [16]. When we restricted the sample to only Nordic-born mothers, the patterns of associations between FER and ASD remained.

While low levels of FER are specific for iron deficiency, high levels are harder to interpret, since they can be a consequence iron overload or part of the acute phase response [25]. If a consequence of iron overload, high levels of FER might indicate increased levels of circulating non-bound iron that may in turn lead to oxidative stress and DNA damage and affect the maternal-placental-fetal unit [60, 61]. There is evidence of u-shaped associations between both maternal hemoglobin and FER levels with obstetric outcomes, such as low birth weight [56]. The increase in FER may also represent increased innate immune activity. The hypoferraemia due to increased concentrations of iron chaperones, such as FER, induced by inflammation is important in the host defense against infection by sequestering iron from pathogens [62]. However, we did not detect any risk associated with high levels of any of the other APP studied to indicate an ongoing increase in innate immune activity.

In a study by Pateva et al., maternal smoking during pregnancy was associated with higher levels of FER and body iron in the mothers, but with reduced FER and iron stores in the newborn children [63]. In contrast, maternal smoking was in our cohort associated with decreased maternal levels of FER in mothers to both cases and controls. The reason for this discrepancy is not clear, but samples in the Pateva study were drawn at the time of birth, whereas the present study used samples drawn in the first trimester, which may have contributed to the discrepant results. Since maternal smoking was not associated with either ASD or any of the stratified outcomes, it was not included as covariate in our adjusted analyses.

Finally, it is important to consider that our observed associations between maternal APP and ASD may not necessarily imply a direct causal involvement of the respective APP, e.g. CRP and FER. Instead, there is a possibility that the abnormal levels might be indicators of some underlying, more general perturbation increasing offspring risk of ASD, that we are not able to capture with our currently available data. Thus, a narrow focus on individual maternal immune markers might not be the most effective approach when considering potential strategies to prevent ASD. Instead, the pursuit of preventive strategies may require a deeper understanding of the normal (beneficial) versus the pathological involvement of the immune system in human neurodevelopment, and the complex interplay between genome, proteome, and environment.

### Strengths and weaknesses

By measuring a range of proteins involved in the maternal innate immune defense in a large population-based cohort, we aimed to increase the likelihood of picking up any signal of maternal immune activation. Further, due to the pleiotropic roles of most APP, we also had the possibility to investigate other pathophysiological processes, not necessarily directly related to immune functioning. We used a validated case-finding procedure [32] and a broad range of register data to control for health- and sociodemographic confounders.

Given that the sample sizes were limited for the stratified analyses, we cannot rule out the presence of subtle associations that we were underpowered to detect in the stratified groups. Hence, we may have missed associations that are indeed present in the population. There is also a possibility that the associations that we do observe in the smaller stratified groups are a result of sampling variability. The coefficient of variation was markedly higher for PCT and tPA compared to the other analytes, and thus the results for PCT and tPA should be interpreted with caution. Although it can be considered a strength to include many immune markers, making multiple statistical comparisons increases the probability of chance findings. Indeed, only the relationship between FER and ASD with ID survived Bonferroni correction, though it should be noted that the Bonferroni adjustment is considered to be conservative [64]. We only have a single measurement at one time-point in pregnancy, restricted to samples drawn in the first trimester for the purpose of interpretability. Consequently, we cannot draw conclusions regarding these markers in later trimesters. Future studies ought to investigate, in more detail, the dynamics of concentrations of APP over the course of pregnancy and their relation to ASD. By restricting our study to samples drawn in the first trimester, we excluded a larger proportion of migrant women and women from low-income families who were not sampled during their first trimester, though a separate sensitivity analysis stratifying the sample on maternal region of birth did not change the estimates.

Since this is an observational study, we cannot give the results a causal interpretation. Although we adjust for a large number of covariates, there is still a possibility that the results are influenced by residual confounding. Maternal region of origin was associated with ASD in a way that is consistent with previous research [65, 66], and also with key markers (CRP and FER) in the study. To control for potential confounding by region of origin, we restricted the cohort to Nordic-born mothers as a sensitivity analysis. A weakness with this strategy is that the highly heterogeneous non-Nordic group was too small to evaluate on their own. However, the associations in the Nordic group, particularly between maternal FER and children’s risk of ASD with ID, were similar to the main analyses, which would not be expected if those associations in the larger population were driven by the underlying association between maternal birth region and both maternal FER level and children’s risk of ASD with ID. There is also the potential for genetic confounding, considering the strong genetic component contributing to population variance of ASD, and that genetic factors also influence levels of several of the APP. If there was an overlap of genetic determinants for ASD and APP, this could potentially result in an association that is not necessarily causal. Finally, there is also a possibility for residual confounding by unmeasured environmental factors that are associated with both changes in the immune system and ASD. For example, maternal exposure to air pollution during pregnancy (measured by particulate matter with diameter ≤ 10 μm (PM10)), has been associated with both measures of innate immunity as well as children’s risk of ASD in some, but not all, studies [67–70].

## Conclusion

We investigated associations between eight maternal inflammatory proteins in first trimester of pregnancy and risk of ASD in offspring. We observed increased odds of ASD without ID or ADHD in offspring to mothers with low levels of CRP, and increased odds of ASD with ADHD in offspring of mothers with elevated levels of CRP and SAP. The biological mechanisms underlying these relationships remain unclear. We also observed increased odds of any ASD, and particularly ASD with ID, in offspring of mothers with both low and elevated levels of FER. Overall, our results give no strong support for the maternal immune activation hypothesis, but rather suggest a more complex relation between maternal immune system markers in early pregnancy varying across specific outcomes. Our results also add support to a plausibly important role of iron metabolism during early neurodevelopment. Future studies ought to investigate the causes of perturbed innate immune signaling in early pregnancy and how this relates to ASD and its comorbidities, and consider measurements of additional biomarkers of iron status to improve our understanding of the relationship between FER and ASD/ID.

## Supplementary information

Supplementary Information
