# Supplementary material for: Maternal levels of acute phase proteins in early pregnancy and risk of autism spectrum disorders in offspring
Source: Transl Psychiatry. 2022 Apr 7;12:148. doi: 10.1038/s41398-022-01907-z (PMC8989993; doi:10.1038/s41398-022-01907-z)
Supplement: Supplementary file 1 — Supplementary Information [file 41398_2022_1907_MOESM1_ESM.docx]

**This files includes Supplementary Figures 1-10 and Supplementary Tables 1-6.**

**Supplementary Figures:**

**Supplementary Figure 1.** Selection of individuals with maternal serum samples.

**Supplementary Figure 2.** Scatterplot and locally weighted regression of two maternal acute phase proteins (C-Reactive Protein and Ferritin) on gestational age at serum sampling.

**Supplementary Figure 3.** Levels of one maternal acute phase protein (Ferritin) according to analytic plate.

**Supplementary Figure 4.** The Spearman rho correlations of eight maternal acute phase proteins with each other, as measured in maternal serum samples of 429 unaffected controls selected from the cohort.

**Supplementary Figure 5.** Heat map showing the mean maternal acute phase protein z-score according to each category of the covariates, among 318 ASD-affected individuals in the cohort.

**Supplementary Figure 6.** The distribution of each acute phase protein measured in maternal serum samples, by ASD-case status.

**Supplementary Figure 7.** The unadjusted relationship between maternal acute phase proteins and odds of ASD, stratified by co-occurrence of ID and ADHD, when comparing 318 ASD cases to 429 unaffected individuals selected from the cohort.

**Supplementary Figure 8.** The relationship between maternal acute phase proteins and odds of any ASD-diagnosis when comparing 318 individuals affected by ASD to 429 unaffected individuals selected from the cohort.

**Supplementary Figure 9.** The relationship between maternal acute phase proteins and odds of ASD in a sensitivity analysis where the study sample is restricted to Nordic-born mothers.

**Supplementary Figure 10.** The relationship between maternal acute phase proteins and odds of ASD in a sensitivity analysis where covariates related to the timing of sampling were included in the model.

**Supplementary Tables:**

**Supplementary Table 1.** Characteristics of individuals in the source population, those for whom neonatal dried blood spots and maternal serum samples were selected, and those for whom samples were selected for acute phase protein analysis.

**Supplementary Table 2.** Quality control statistics for multiplex assays to analyze acute phase protein concentrations in maternal serum samples.

**Supplementary Table 3.** Characteristics of individuals diagnosed with ASD and unaffected individuals in the study sample.

**Supplementary Table 4.** P-values for the association of maternal acute phase proteins with other covariates among mothers to 429 unaffected individuals.

**Supplementary Table 5.** P-values for the association of maternal acute phase proteins with other covariates among mothers to 318 ASD-affected individuals.

**Supplementary Table 6.** P-values for non-linearity in restricted cubic spline analyses.

**Supplementary Figure 1.**Selection of individuals with maternal serum samples from the Stockholm Youth Cohort (SYC) source population.

*A random sample of SYC that includes individuals with ASD also included among the total ASD cases sampled.

** Diagnostic update as of 2016-12-31, that resulted in 29 incident cases among unaffected siblings to ASD cases, and 40 incident cases among the ASD-unaffected control individuals.

**Supplementary Figure 2.** Scatterplot and locally weighted regression of C-Reactive Protein (CRP) (A) and Ferritin (FER) (B) on gestational age at serum sampling.


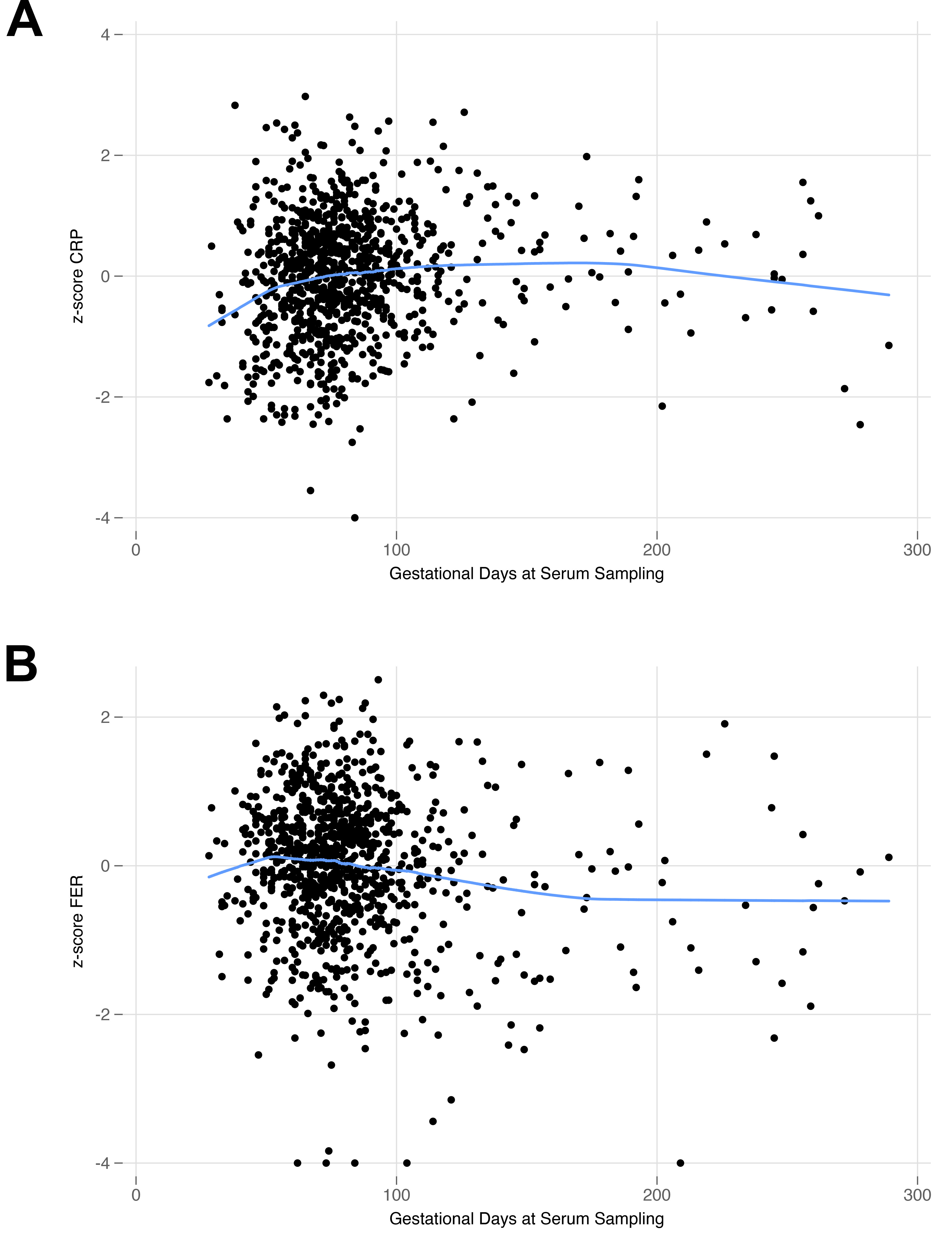


**Supplementary Figure 3.** Boxplots of log-2-values of Ferritin (FER) over the 13 assay plates (A).  Z-scores of FER over the 13 assay plates (B). Kernel density plot of Z-scores of FER with tertile cut-points for categorical analysis (C).

**Supplementary Figure 4.** The Spearman rho correlations of eight APP with each other, as measured in maternal serum samples of 429 unaffected controls. Abbreviations **A2M**: α-2-macroglobulin; **CRP**: C-reactive protein; **FER**: ferritin; **FIB**: fibrinogen; **PCT**: procalcitonin; **SAA**: serum amyloid A; **SAP**: serum amyloid P; and **tPA**: tissue plasminogen activator.


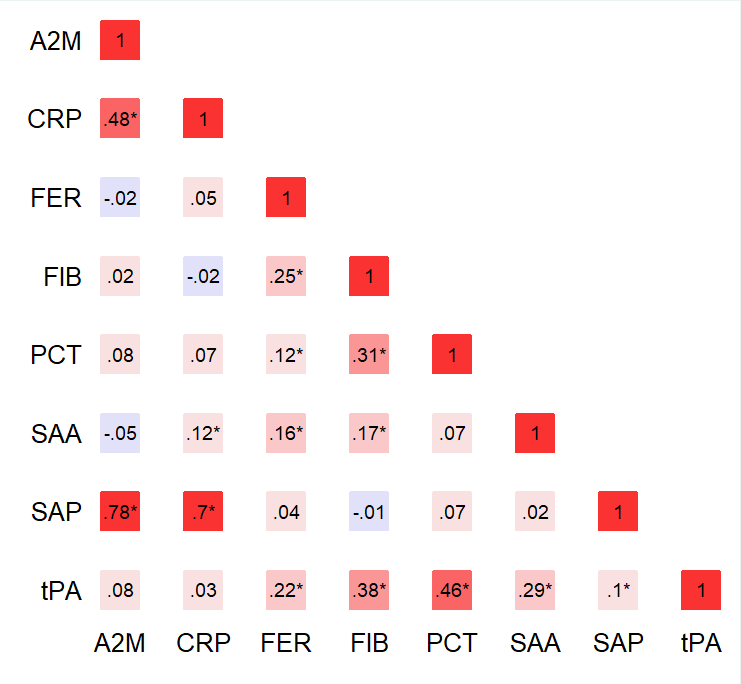


**Supplementary Figure 5.** Heat map showing the mean APP z-score by categories of the covariates, among 318 ASD-affected individuals in the cohort. Solid boxes indicate that the APP is associated with the covariate at p<0.05. Dashed boxes indicate that the APP is associated with the covariate at p<0.20. Abbreviations: **A2M**: α-2 macroglobulin; **CRP**: C-reactive protein; **FER**: ferritin; **FIB**: fibrinogen; **PCT**: procalcitonin; **SAA**: serum amyloid A; **SAP**: serum amyloid P; **tPA**: tissue plasminogen activator; Psych: Psychiatric; BMI: body mass index; Income Q: income quintile.


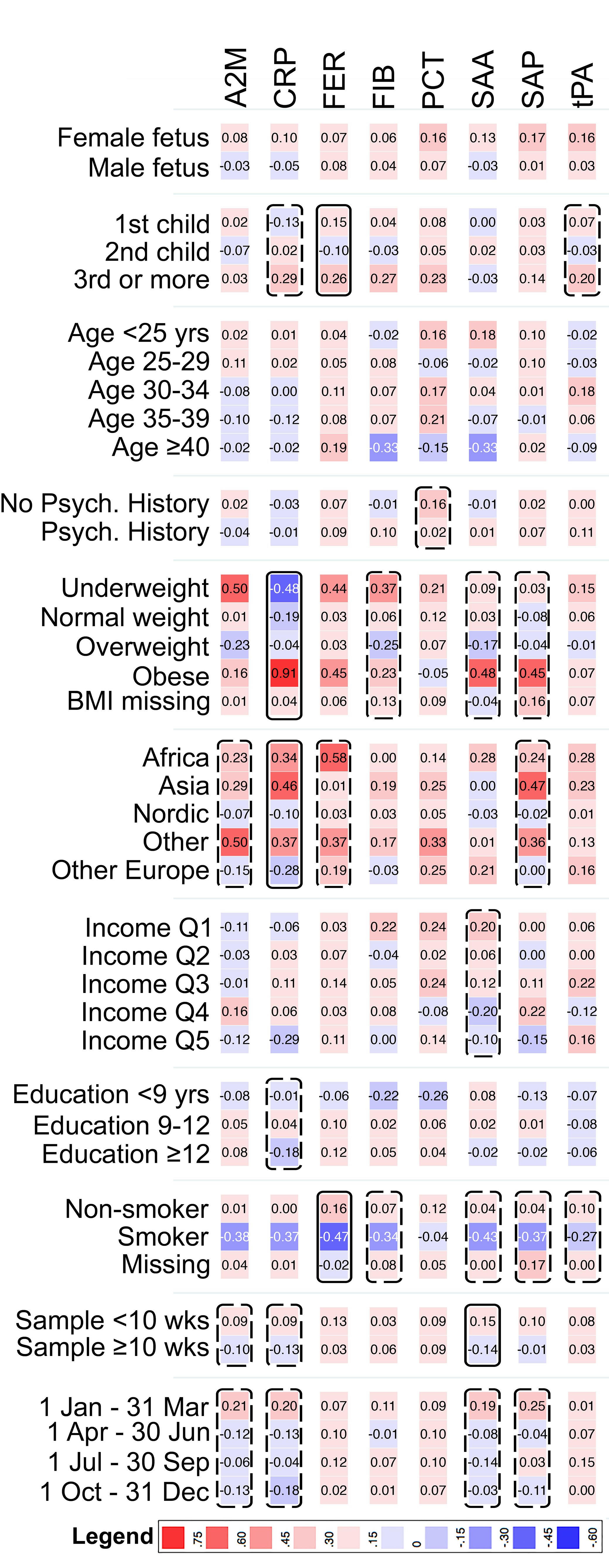


**Supplementary Figure 6**. Distribution of each APP measured in maternal serum samples, by ASD-case status.

**
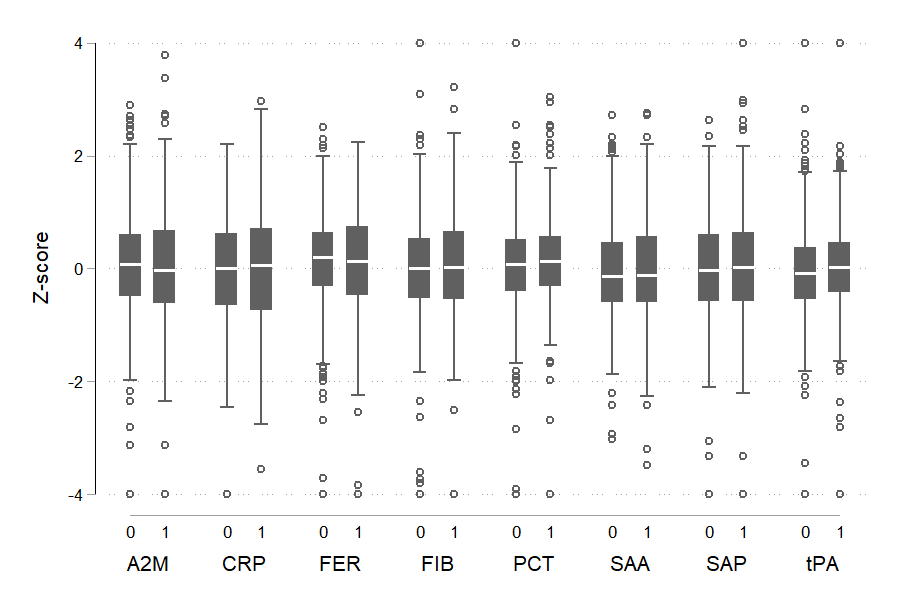
**

**Supplementary Figure 7**. The unadjusted relationship between APP and odds of ASD, stratified by co-occurrence of ID and ADHD, when comparing 318 ASD cases to 429 unaffected individuals selected from the cohort. Tertiles of each APP were created using the distribution of z-scores among unaffected individuals to set the cut-offs and the middle tertile was used as the referent category. Error bars represent the 95% confidence interval. P-values are shown for a Wald test with a null hypothesis that all APP categorical terms were jointly equal to zero, as a test of whether each APP was generally associated with the outcome. Abbreviations: **A2M**: α-2 macroglobulin; **CRP**: C-reactive protein; **FER**: ferritin; **FIB**: fibrinogen; **PCT**: procalcitonin; **SAA**: serum amyloid A; **SAP**: serum amyloid P; and **tPA**: tissue plasminogen activator.

**
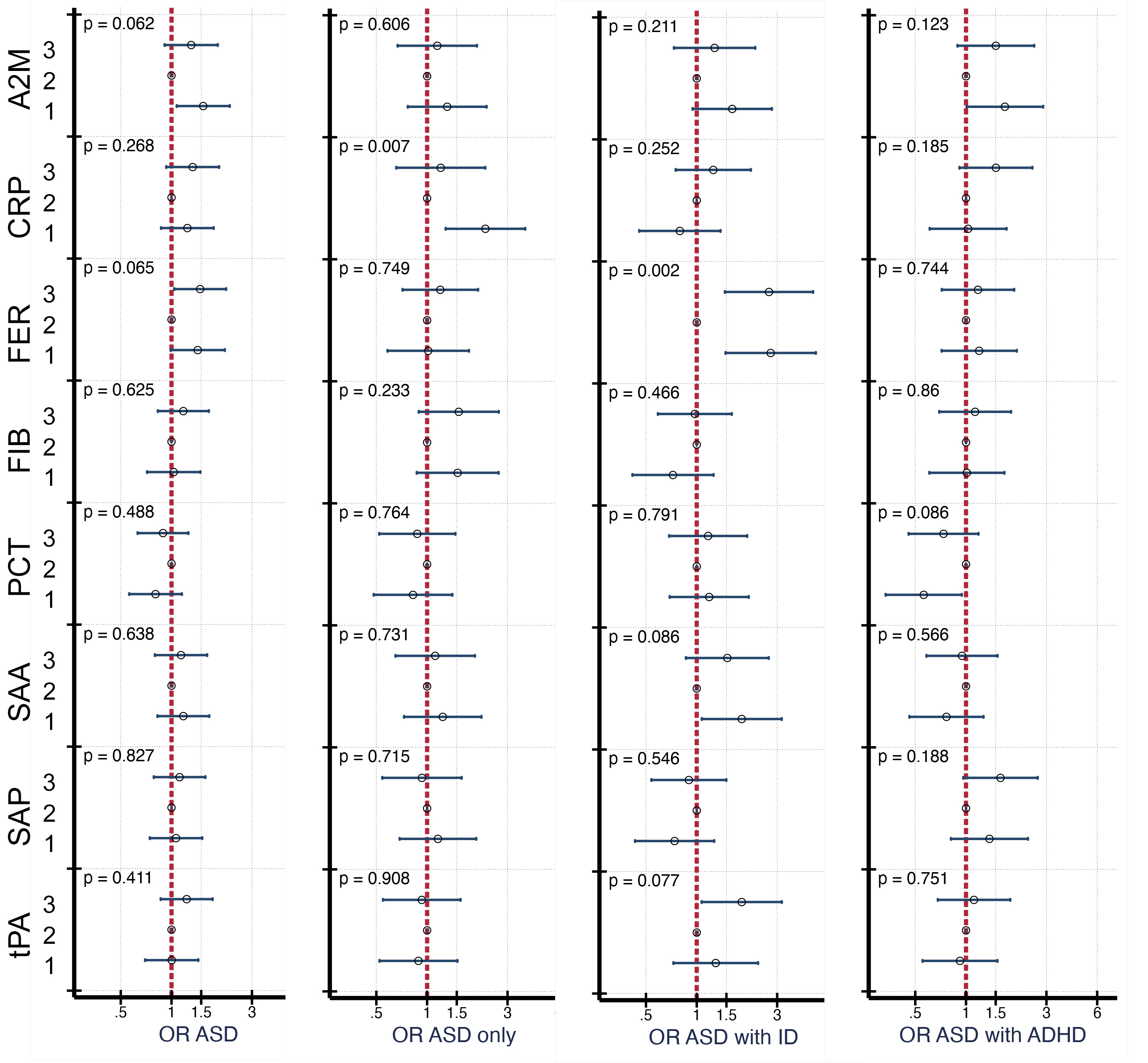
**

**Supplementary Figure 8.** The relationship between APP and odds of ASD when comparing 318 individuals affected by any ASD-diagnosis to 429 unaffected individuals selected from the cohort. Each panel displays the odds of ASD according to APP z-score, flexibly fit using restricted cubic spline models with three knots and a z-score=0 as the referent. The dashed line represents the unadjusted estimate of the relationship between each APP and odds of ASD. The solid line represents the fully adjusted model, adjusted for sex, birth order, maternal BMI, maternal psychiatric history, maternal region of origin, maternal age and family income quintile. The gray bands represent the 95% confidence interval for the fully adjusted model. P-values are shown for a Wald test with a null hypothesis that all APP spline terms were jointly equal to zero, as a test of whether each APP was generally associated with the outcome. Abbreviations: **A2M**: α-2 macroglobulin; **CRP**: C-reactive protein; **FER**: ferritin; **FIB**: fibrinogen; **PCT**: procalcitonin; **SAA**: serum amyloid A; **SAP**: serum amyloid P; and **tPA**: tissue plasminogen activator.


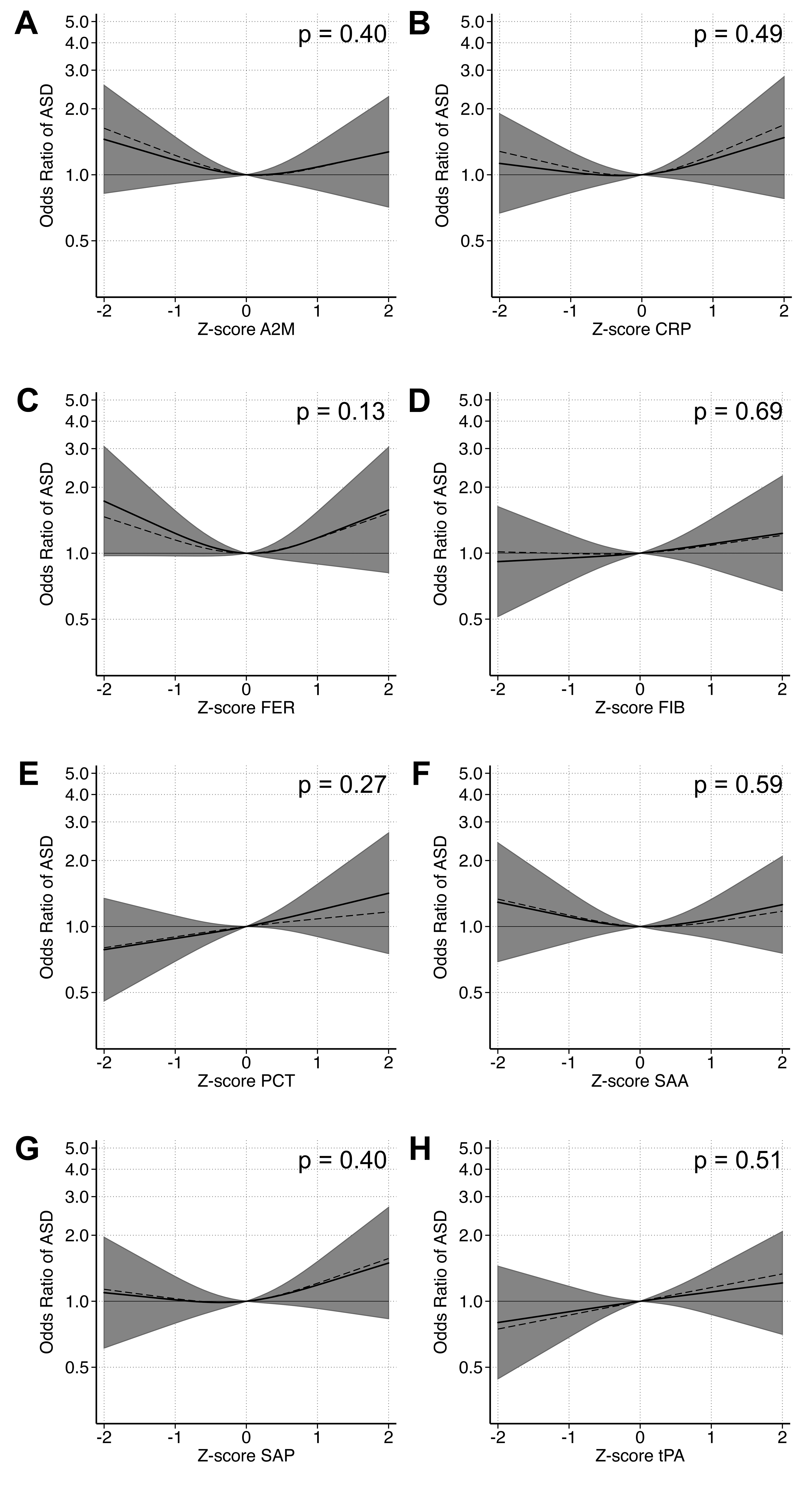


**Supplementary Figure 9.** The relationship between APP and odds of ASD, stratified by co-occurrence of ID and ADHD, when comparing 250 ASD cases to 349 unaffected individuals selected from the cohort. As a sensitivity analysis, the cohort was restricted to Nordic-born mothers. Tertiles of each APP were created using the distribution of z-scores among unaffected individuals to set the cut-offs and the middle tertile was used as the referent category. Models were adjusted for sex, birth order, maternal BMI, maternal psychiatric history, maternal region of origin, maternal age and family income quintile. Error bars represent the 95% confidence interval. P-values are shown for a Wald test with a null hypothesis that all APP categorical terms were jointly equal to zero, as a test of whether each APP was generally associated with the outcome. Abbreviations: **A2M**: α-2 macroglobulin; **CRP**: C-reactive protein; **FER**: ferritin; **FIB**: fibrinogen; **PCT**: procalcitonin; **SAA**: serum amyloid A; **SAP**: serum amyloid P; and **tPA**: tissue plasminogen activator.


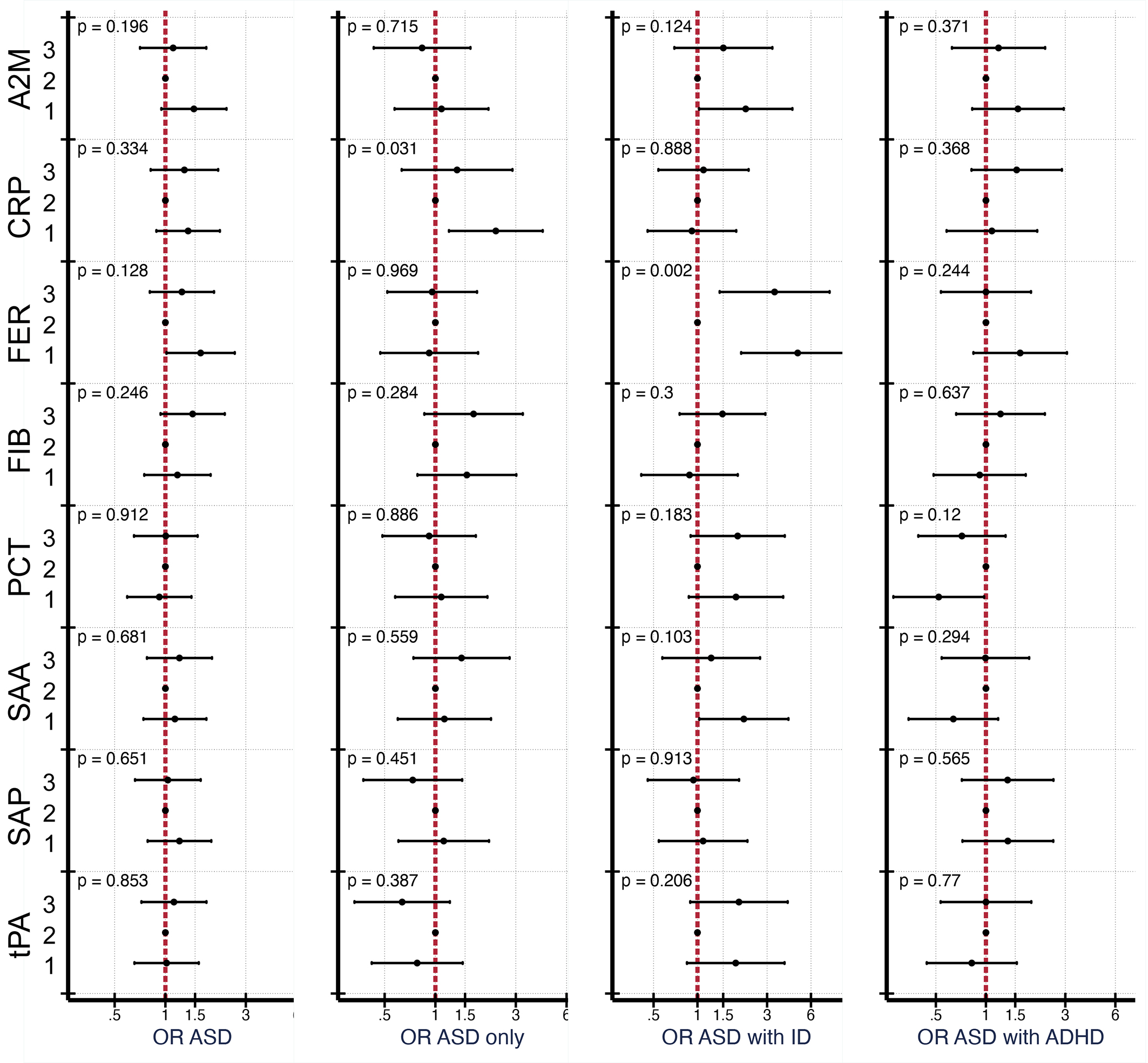


**Supplementary Figure 10.** The relationship between APP and odds of ASD, stratified by co-occurrence of ID and ADHD, when comparing 250 ASD cases to 349 unaffected individuals selected from the cohort. As a sensitivity analysis, regression models were adjusted for annual quarter at serum sample and gestational week at serum sample, in addition to covariates in the main analysis. Tertiles of each APP were created using the distribution of z-scores among unaffected individuals to set the cut-offs and the middle quintile was used as the referent category. Error bars represent the 95% confidence interval. P-values are shown for a Wald test with a null hypothesis that all APP categorical terms were jointly equal to zero, as a test of whether each APP was generally associated with the outcome. Abbreviations: **A2M**: α-2 macroglobulin; **CRP**: C-reactive protein; **FER**: ferritin; **FIB**: fibrinogen; **PCT**: procalcitonin; **SAA**: serum amyloid A; **SAP**: serum amyloid P; and **tPA**: tissue plasminogen activator.


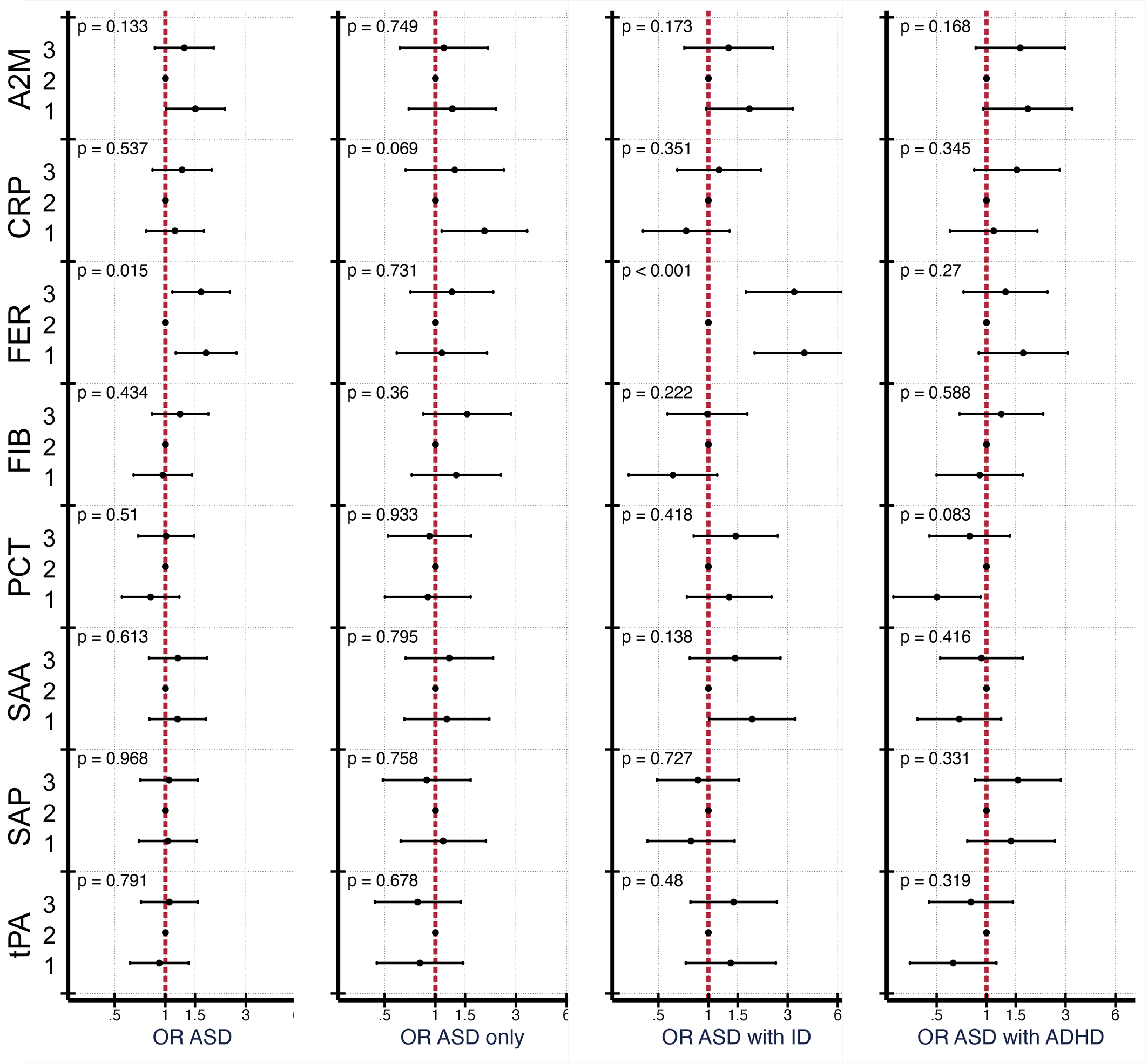


**Supplementary Table 1.** Characteristics of individuals in the source population (SYC Source Population), those for whom neonatal dried blood spots were selected (NDBS Cohort), those in NDBS cohort who were selected for APP-analysis (NDBS APP Cohort), those for whom maternal serum samples were selected (MS Cohort), and those for whom maternal serum samples were selected for APP analysis (MS Analysis Cohort).

|  |  | *SYC Source Cohort^1^* | *NDBS Cohort^2^* | *NDBS APP Cohort^3^* | *MS Cohort^4^* | *MS Analysis Cohort^5^* |
| --- | --- | --- | --- | --- | --- | --- |
|  |  | **N = 98 597** | **N = 1 847** | **N = 1 154** | **N=559** | **N=438** |
| Sex | | | | |  |  |
|  | Female | 48 158 (48.8%) | 879 (47.6%) | 556 (48.2%) | 259 (46.3%) | 206 (47.0%) |
|  | Male | 50 439 (51.2%) | 968 (52.4%) | 598 (51.8%) | 300 (53.7%) | 232 (53.0%) |
| Birth Order | | | | |  |  |
|  | 1st born | 44 178 (44.8%) | 810 (43.9%) | 505 (43.8%) | 242 (43.2%) | 192 (43.8%) |
|  | 2nd born | 36 238 (36.8%) | 677 (36.7%) | 426 (36.9%) | 216 (38.6%) | 171 (39.0%) |
|  | 3rd or higher | 18 030 (18.3%) | 360 (19.9%) | 223 (19.3%) | 101 (18.0%) | 75 (17.1%) |
|  | missing | 151 (0.2%) |  |  |  |  |
| Maternal Age (years) | | | | |  |  |
|  | >25 | 13 767 (14.0%) | 251 (13.6%) | 156 (13.5%) | 66 (11.8%) | 45 (10.3%) |
|  | 25-29 | 29 684 (30.1%) | 572 (31.0%) | 322 (27.9%) | 149 (26.7%) | 112 (25.6%) |
|  | 30-34 | 35 526 (36.0%) | 634 (34.3%) | 406 (35.2%) | 218 (39.0%) | 179 (40.9%) |
|  | 35-39 | 16 302 (16.5%) | 336 (18.2%) | 235 (20.4%) | 112 (20.0%) | 90 (20.5%) |
|  | ≥40 | 3 161 (3.2%) | 54 (2.9%) | 35 (3.0%) | 14 (2.5%) | 12 (2.7%) |
|  | missing | 157 (0.2%) |  |  |  |  |
| Maternal Psychiatric History | | | | |  |  |
|  | No | 65 155 (66.1%) | 1 210 (65.5%) | 756 (65.5%) | 380 (68.0%) | 292 (66.7%) |
|  | Yes | 33 288 (33.8%) | 637 (34.5%) | 398 (34.5%) | 179 (32.0%) | 146 (33.3%) |
|  | missing | 154 (0.2%) |  |  |  |  |
| Maternal BMI (kg/m^2^) | | | | |  |  |
|  | Normal | 49 181 (49.9%) | 935 (50.6%) | 564 (48.9%) | 265 (47.4%) | 216 (49.3%) |
|  | Underweight | 2252 (2.3%) | 39 (2.1%) | 23 (2.0%) | 9 (1.6%) | 8 (1.8%) |
|  | Overweight | 15 084 (15.3%) | 308 (16.7%) | 197 (17.1%) | 85 (15.2%) | 63 (14.4%) |
|  | Obese | 4 780 (4.8%) | 99 (5.4%) | 52 (4.5%) | 24 (4.3%) | 16 (3.7%) |
|  | missing | 27 300 (27.7%) | 466 (25.2%) | 318 (27.6%) | 176 (31.5%) | 135 (30.8%) |
| Maternal Region of Birth | | | | |  |  |
|  | Africa | 4 807 (4.9%) | 103 (5.6%) | 67 (5.8%) | 33 (5.9%) | 18 (4.1%) |
|  | Asia | 10 960 (11.1%) | 186 (10.1%) | 114 (9.9%) | 55 (9.8%) | 34 (7.8%) |
|  | Nordic | 76 086 (77.2%) | 1 430 (77.4%) | 888 (76.9%) | 430 (76.9%) | 357 (81.5%) |
|  | Other | 2 523 (2.6%) | 52 (2.8%) | 38 (3.3%) | 20 (3.6%) | 16 (3.7%) |
|  | Other Europe | 4 067 (4.1%) | 76 (4.1%) | 47 (4.1%) | 21 (3.8%) | 13 (3.0%) |
|  | missing | 154 (0.16%) |  |  |  |  |
| Family Income Quintile | | | | |  |  |
|  | 1 | 14 221 (14.4%) | 267 (14.5%) | 161 (14.0%) | 67 (12.0%) | 42 (9.6%) |
|  | 2 | 20 209 (20.5%) | 388 (21.0%) | 248 (21.5%) | 115 (20.6%) | 79 (18.0%) |
|  | 3 | 21 187 (21.5%) | 398 (21.5%) | 253 (21.9%) | 108 (19.3%) | 92 (21.0%) |
|  | 4 | 21 518 (21.8%) | 390 (21.1%) | 249 (21.6%) | 124 (22.2%) | 100 (22.8%) |
|  | 5 | 21 444 (21.7%) | 391 (21.2%) | 243 (21.1%) | 145 (25.9%) | 125 (28.5%) |
|  | missing | 18 (<1%) | 13 (0.7%) |  |  |  |
| Maternal Education Level at Birth | | | | |  |  |
|  | <9 years | 15 302 (15.5%) | 280 (15.2%) | 175 (15.2%) | 84 (15.0%) | 56 (12.8%) |
|  | 9-12 years | 44 067 (44.7%) | 820 (44.4%) | 501 (43.4%) | 230 (41.1%) | 186 (42.5%) |
|  | >12 | 38 647 (39.2%) | 727 (39.4%) | 475 (41.2%) | 242 (43.3%) | 195 (44.5%) |
|  | missing | 581 (0.6%) | 20 (1.1%) | 3 (0.3%) | 3 (0.5%) | 1 (0.2%) |
| Trimester at First Antenatal Visit | | | | | | |
|  | 1 | 64 220 (65.1%) | 1 351 (73.1%) | 856 (74.2%) | 466 (83.4%) | 438 (100.0%) |
|  | 2 | 11 862 (12.0%) | 256 (13.9%) | 173 (15.0%) | 82 (14.7%) |  |
|  | 3 | 1 246 (1.3%) | 22 (1.2%) | 15 (1.3%) | 10 (1.8%) |  |
|  | missing | 21 269 (21.6%) | 218 (11.8%) | 110 (9.5%) | 1 (0.2%) |  |
| Number of Antenatal Visits | | | | | | |
|  | <9 | 14 362 (14.6%) | 270 (14.6%) | 189 (16.4%) | 95 (17.0%) | 54 (12.3%) |
|  | 9-13 | 47 819 (48.5%) | 909 (49.2%) | 589 (51.0%) | 278 (49.7%) | 238 (54.3%) |
|  | ≥14 | 8 613 (8.7%) | 158 (8.6%) | 81 (7.0%) | 30 (5.4%) | 28 (6.4%) |
|  |  | 27 803 (28.2%) | 510 (27.6%) | 295 (25.6%) | 156 (27.9%) | 1. 6.9%) |

1. Cohort selected from the Stockholm Youth Cohort (SYC), from those born in Sweden between 1996 and 2000, and resident in Stockholm County for at least four years. See Supplementary Figure 1 for corresponding flowchart of sample selection.
2. Individuals selected for collection of neonatal dried blood spot (NDBS) sample as a random sample from the larger SYC cohort. See Supplementary Figure 1.
3. Individuals selected for analysis of acute phase proteins in NDBS samples. See Supplementary Figure 1.
4. Individuals for whom maternal serum samples were collected. See Supplementary Figure 1.
5. Individuals for whom maternal serum samples were selected for analysis of acute phase proteins. See Supplementary Figure 1.

**Supplementary Table 2.** Quality control statistics for multiplex assays to analyze acute phase protein concentrations in maternal serum samples.

| Analyte | Inter-Assay %CV (controls)^1^ | Intra-Assay %CV (controls)^1^ | Below LLOQ | LLOQ^2^ | Above ULOQ | ULOQ^3^ | Imputed^4^ |
| --- | --- | --- | --- | --- | --- | --- | --- |
| α-2-macroglobulin | 15.8 | 4.93 | 3 (0.40%) | 0.61 ng/ml | 3 (0.40%) | 4062.09 ng/ml | 6 (0.80%) |
| C Reactive Protein | 13.9 | 7.68 | 3 (0.40%) | 0.01 ng/ml | 1 (0.13%) | 57 ng/ml | 4 (0.54%) |
| Ferritin | 20.5 | 10.68 | 3 (0.40%) | 2.49 pg/ml | 0 (0.00%) | 39405.2 pg/ml | 3 (0.40%) |
| Fibrinogen | 11.2 | 5.99 | 4 (0.54%) | 3.01 ng/ml | 0 (0.00%) | 758.4 ng/ml | 4 (0.54%) |
| Haptoglobin | 16.2 | 6.09 | 2 (0.27%) | 0.04 ng/ml | 564 (75.50%) | 277.86 ng/ml | 566 (75.77%) |
| Procalcitonin | 34.5 | 16.28 | 5 (0.67%) | 4.4 pg/ml | 0 (0.00%) | 5365.04 pg/ml | 5 (0.67%) |
| Serum Amyloid A | 18.7 | 10.54 | 5 (0.67%) | 0.29 ng/ml | 118 (15.80%) | 388.44 ng/ml | 123 (16.47%) |
| Serum Amyloid P | 11.4 | 4.12 | 3 (0.40%) | 0.01 ng/ml | 36 (4.82%) | 186.54 ng/ml | 39 (5.22%) |
| Tissue Plasminogen Activator | 25.5 | 10.79 | 4 (0.54%) | 1.95 pg/ml | 0 (0.00%) | 4487.54 pg/ml | 4 (0.54%) |
| Average | 18.6 | 8.6 | 0.48% |  | 10.74% |  | 11.21% |

1. The percent coefficients of variation (CV) are based on standardized controls that were run in duplicate on each of the 13 assay plates.
2. Lower Limit of Quantitation, average across 13 assay plates.
3. Upper Limit of Quantitation, average across 13 assay plates.
4. The absolute and relative frequency of observations that were imputed, including those values that were below the LLOQ, above the ULOQ, or that the Bio-plex Manager software indicated were near the LLOQ and thus less certain than values higher in the range of quantitation.

**Supplementary Table 3.** Characteristics of individuals diagnosed with ASD and unaffected individuals in the study sample.

|  | Unaffected  (n = 429) | ASD  (n = 318) | p-value^1^ | ASD only  (n = 100) | ASD with ID  (n = 101) | ASD with ADHD  (n = 117) | p-value^2^ |
| --- | --- | --- | --- | --- | --- | --- | --- |
| Sex | | | | | | | |
| Female | 204 (47.6%) | 69 (21.7%) | <0.001 | 19 (19.0%) | 27 (26.7%) | 23 (19.7%) | <0.001 |
| Male | 225 (52.4%) | 249 (78.3%) |  | 81 (81.0%) | 74 (73.3%) | 94 (80.3%) |  |
| Birth Order | | | | | | | |
| 1st born | 187 (43.6%) | 164 (51.6%) | 0.090 | 57 (57.0%) | 40 (39.6%) | 67 (57.3%) | 0.031 |
| 2nd born | 167 (38.9%) | 109 (34.3%) |  | 30 (30.0%) | 45 (44.6%) | 34 (29.1%) |  |
| 3rd or higher | 75 (17.5%) | 45 (14.2%) |  | 13 (13.0%) | 16 (15.8%) | 16 (13.7%) |  |
| Maternal Age (Years) | | | | | | | |
| < 25 | 43 (10.0%) | 38 (11.9%) | 0.041 | 8 (8.0%) | 12 (11.9%) | 18 (15.4%) | 0.054 |
| 25-29 | 108 (25.2%) | 101 (31.8%) |  | 29 (29.0%) | 27 (26.7%) | 45 (38.5%) |  |
| 30-34 | 178 (41.5%) | 101 (31.8%) |  | 34 (34.0%) | 37 (36.6%) | 30 (25.6%) |  |
| 35-39 | 89 (20.7%) | 64 (20.1%) |  | 24 (24.0%) | 19 (18.8%) | 21 (17.9%) |  |
| ≥ 40 | 11 (2.6%) | 14 (4.4%) |  | 5 (5.0%) | 6 (5.9%) | 3 (2.6%) |  |
| Maternal Psychiatric History | | | | | | | |
| No | 289 (67.4%) | 162 (50.9%) | <0.001 | 50 (50.0%) | 60 (59.4%) | 52 (44.4%) | <0.001 |
| Yes | 140 (32.6%) | 156 (49.1%) |  | 50 (50.0%) | 41 (40.6%) | 65 (55.6%) |  |
| Maternal BMI | | | | | | | |
| Underweight | 8 (1.9%) | 7 (2.2%) | 0.057 | 2 (2.0%) | 3 (3.0%) | 2 (1.7%) | 0.005 |
| Normal | 213 (49.7%) | 126 (39.6%) |  | 49 (49.0%) | 43 (42.6%) | 34 (29.1%) |  |
| Overweight | 59 (13.8%) | 54 (17.0%) |  | 11 (11.0%) | 18 (17.8%) | 25 (21.4%) |  |
| Obese | 16 (3.7%) | 21 (6.6%) |  | 2 (2.0%) | 6 (5.9%) | 13 (11.1%) |  |
| Missing | 133 (31.0%) | 110 (34.6%) |  | 36 (36.0%) | 31 (30.7%) | 43 (36.8%) |  |
| Maternal Country of Birth | | | | | | | |
| Africa | 18 (4.2%) | 16 (5.0%) | 0.89 | 3 (3.0%) | 12 (11.9%) | 1 (0.9%) | <0.001 |
| Asia | 34 (7.9%) | 27 (8.5%) |  | 8 (8.0%) | 18 (17.8%) | 1 (0.9%) |  |
| Nordic | 349 (81.4%) | 250 (78.6%) |  | 83 (83.0%) | 61 (60.4%) | 106 (90.6%) |  |
| Other | 15 (3.5%) | 12 (3.8%) |  | 4 (4.0%) | 4 (4.0%) | 4 (3.4%) |  |
| Other Europe | 13 (3.0%) | 13 (4.1%) |  | 2 (2.0%) | 6 (5.9%) | 5 (4.3%) |  |
| Family Income Quintile | | | | | | | |
| 1^st^ (Lowest) | 41 (9.6%) | 39 (12.3%) | 0.006 | 8 (8.0%) | 22 (21.8%) | 9 (7.7%) | <0.001 |
| 2^nd^ | 75 (17.5%) | 81 (25.5%) |  | 20 (20.0%) | 28 (27.7%) | 33 (28.2%) |  |
| 3^rd^ | 90 (21.0%) | 64 (20.1%) |  | 22 (22.0%) | 17 (16.8%) | 25 (21.4%) |  |
| 4^th^ | 100 (23.3%) | 75 (23.6%) |  | 18 (18.0%) | 23 (22.8%) | 34 (29.1%) |  |
| 5^th^ | 123 (28.7%) | 59 (18.6%) |  | 32 (32.0%) | 11 (10.9%) | 16 (13.7%) |  |
| Maternal Education at Birth | | | | | | | |
| < 9 years | 55 (12.8%) | 42 (13.2%) | 0.22 | 10 (10.0%) | 13 (12.9%) | 19 (16.2%) | 0.14 |
| 9-12 years | 179 (41.7%) | 151 (47.5%) |  | 41 (41.0%) | 52 (51.5%) | 58 (49.6%) |  |
| > 12 years | 194 (45.2%) | 124 (39.0%) |  | 49 (49.0%) | 35 (34.7%) | 40 (34.2%) |  |
| missing | 1 (0.2%) | 1 (0.3%) |  |  | 1 (1.0%) |  |  |
| Gestational Week at Serum Sample | | | | | | | |
| < 10 weeks | 203 (47.3%) | 160 (50.3%) | 0.42 | 52 (52.0%) | 48 (47.5%) | 60 (51.3%) | 0.77 |
| 10-13 weeks | 226 (52.7%) | 158 (49.7%) |  | 48 (48.0%) | 53 (52.5%) | 57 (48.7%) |  |
| Serum Sampling Quarter | | | | | | | |
| 1 January - 31 March | 126 (29.4%) | 98 (30.8%) | 0.33 | 30 (30.0%) | 35 (34.7%) | 33 (28.2%) | 0.54 |
| 1 April - 30 June | 101 (23.5%) | 90 (28.3%) |  | 34 (34.0%) | 24 (23.8%) | 32 (27.4%) |  |
| 1 July - 30 September | 96 (22.4%) | 64 (20.1%) |  | 17 (17.0%) | 23 (22.8%) | 24 (20.5%) |  |
| 1 October - 31 December | 106 (24.7%) | 66 (20.8%) |  | 19 (19.0%) | 19 (18.8%) | 28 (23.9%) |  |
| Smoking at First Antenatal Visit | | | | | | | |
| No | 297 (69.2%) | 220 (69.2%) | 1.00 | 67 (67.0%) | 74 (73.3%) | 79 (67.5%) | 0.31 |
| Yes | 27 (6.3%) | 20 (6.3%) |  | 3 (3.0%) | 5 (5.0%) | 12 (10.3%) |  |
| Missing | 105 (24.5%) | 78 (24.5%) |  | 30 (30.0%) | 22 (21.8%) | 26 (22.2%) |  |
| Proteins measured in maternal serum samples (median [IQR]) | | | | | | | |
| A2M (mg/ml) | 2.40 (1.60, 3.78) | 2.24 (1.55, 3.71) | 0.48 | 2.24 (1.58, 3.35) | 2.19 (1.66, 4.15) | 2.25 (1.45, 3.77) | 0.77 |
| CRP (µg/ml) | 9.35 (3.96, 29.97) | 9.73 (3.31, 3.68) | 0.80 | 5.81 (2.86, 2.11) | 13.04 (5.10, 41.30) | 13.47 (3.85, 40.51) | 0.040 |
| FER (ng/ml) | 85.39 (47.35, 148.83) | 89.23 (44.03, 162.51) | 0.84 | 93.08 (53.00, 170.73) | 90.14 (39.11, 159.91) | 82.74 (43.82, 155.07) | 0.63 |
| FIB (ng/ml) | 3.72 (3.18, 4.47) | 3.76 (3.16, 4.59) | 0.70 | 3.81 (3.15, 4.63) | 3.75 (3.28, 4.61) | 3.77 (3.14, 4.55) | 0.86 |
| PCT (ng/ml) | 6.56 (5.01, 8.19) | 6.63 (5.07, 8.25) | 0.66 | 6.81 (5.02, 8.55) | 6.36 (5.15, 7.69) | 6.77 (5.16, 8.25) | 0.80 |
| SAA (ng/ml) | 9.58 (5.12, 18.35) | 9.06 (4.62, 19.60) | 0.65 | 8.30 (4.71, 17.53) | 8.01 (4.16, 23.04) | 10.37 (5.27, 19.46) | 0.75 |
| SAP (µg/ml) | 120.50 (64.38, 332.40) | 122.61 (64.66, 336.81) | 0.71 | 99.23 (64.76, 232.21) | 152.39 (73.19, 326.99) | 135.06 (55.28, 389.28) | 0.44 |
| tPA (ng/ml) | 6.52 (4.89, 8.50) | 6.54 (5.02, 9.07) | 0.46 | 6.24 (4.89, 8.98) | 6.89 (5.54, 9.13) | 6.46 (4.91, 8.94) | 0.75 |

1. Pearson’s chi-squared test was used for categorical variables, comparing the frequency distributions among unaffected individuals to the distributions among all ASD-affected individuals. Kruskal-Wallis tests were used for continuous variables, as the distributions of the APP concentrations were strongly skewed.
2. Pearson’s chi-squared test was used for categorical variables, comparing the frequency distributions among unaffected individuals to the distribution among the stratified ASD outcome groups. Kruskal-Wallis tests were used for continuous variables, as the distributions of the APP concentrations were strongly skewed.

Abbreviations: **ASD**: autism spectrum disorders; **ADHD**: attention-deficit/hyperactivity disorder; **ID**: intellectual disability; **BMI**: body mass index; **IQR**: interquartile range; **A2M**: α-2 macroglobulin; **CRP**: C reactive protein; **FER**: ferritin; **FIB**: fibrinogen; **PCT**: procalcitonin; **SAA**: serum amyloid A; **SAP**: serum amyloid P; and **tPA**: tissue plasminogen activator.

**Supplementary Table 4**. P-values for the association of maternal APP with other covariates among 429 unaffected individuals. We examined the association of each covariate with the APP z-scores by regressing each APP over the categories of the covariates, followed by a joint Wald test of the hypothesis that all coefficients for the categorical indicators are equal to zero, a test whether each APP was generally associated with the covariate.

|  | *A2M* | *CRP* | *FER* | *FIB* | *PCT* | *SAA* | *SAP* | *tPA* | |
| --- | --- | --- | --- | --- | --- | --- | --- | --- | --- |
| *Sex* | 0.169 | 0.287 | 0.169 | 0.323 | 0.724 | 0.705 | 0.690 | 0.396 |  |
| *Birth Order* | **0.023** | 0.166 | 0.354 | 0.424 | 0.267 | 0.278 | 0.072 | 0.162 |  |
| *Maternal Age* | 0.250 | 0.165 | 0.329 | 0.492 | 0.256 | 0.146 | 0.307 | 0.212 |  |
| *Maternal Psych. Hist.* | 0.525 | 0.669 | 0.330 | 0.311 | 0.637 | **0.029** | 0.983 | 0.190 |  |
| *Maternal BMI* | **0.049** | **<0.001** | 0.840 | 0.583 | 0.320 | 0.259 | **0.039** | 0.540 |  |
| *Maternal Country of Birth* | 0.786 | 0.163 | 0.129 | 0.055 | 0.165 | 0.683 | 0.471 | 0.257 |  |
| *Parental Income* | 0.740 | 0.131 | 0.225 | 0.532 | 0.088 | 0.399 | 0.571 | 0.054 |  |
| *Maternal Education at Birth* | 0.512 | 0.087 | 0.434 | 0.142 | 0.061 | 0.801 | 0.604 | 0.986 |  |
| *Maternal Smoking* | 0.262 | 0.755 | 0.112 | 0.220 | 0.198 | 0.151 | 0.439 | 0.547 |  |
| *Gest. Age at Sampling* | 0.276 | **0.001** | 0.852 | 0.502 | 0.896 | 0.660 | 0.189 | 0.975 |  |
| *Sampling Quarter* | 0.817 | 0.745 | 0.473 | 0.551 | 0.989 | 0.916 | 0.996 | 0.224 |  |

Abbreviations: **Psych**: Psychiatric; **Hist**; History; **BMI**: Body Mass Index; **A2M**: α-2 macroglobulin; **CRP**: C-reactive protein; **FER**: ferritin; **FIB**: fibrinogen; **PCT**: procalcitonin; **SAA**: serum amyloid A; **SAP**: serum amyloid P; and **tPA**: tissue plasminogen activator.

**Supplementary Table 5**. P-values for the association of maternal APP with other covariates among 318 ASD affected individuals. We examined the association of each covariate with the APP z-scores by regressing each APP over the categories of the covariates, followed by a joint Wald test of the hypothesis that all coefficients for the categorical indicators are equal to zero, a test whether each APP was generally associated with the covariate.

|  | *A2M* | *CRP* | *FER* | *FIB* | *PCT* | *SAA* | *SAP* | *tPA* | |
| --- | --- | --- | --- | --- | --- | --- | --- | --- | --- |
| *Sex* | 0.393 | 0.308 | 0.954 | 0.897 | 0.502 | 0.253 | 0.265 | 0.302 |  |
| *Birth Order* | 0.710 | 0.053 | **0.043** | 0.173 | 0.511 | 0.951 | 0.802 | 0.359 |  |
| *Maternal Age* | 0.656 | 0.942 | 0.973 | 0.579 | 0.228 | 0.528 | 0.941 | 0.497 |  |
| *Maternal Psych. Hist.* | 0.563 | 0.832 | 0.833 | 0.276 | 0.177 | 0.848 | 0.622 | 0.334 |  |
| *Maternal BMI* | 0.280 | **<0.001** | 0.340 | 0.084 | 0.944 | 0.161 | 0.116 | 0.984 |  |
| *Maternal Country of Birth* | 0.108 | **0.025** | 0.172 | 0.894 | 0.646 | 0.754 | 0.092 | 0.619 |  |
| *Parental Income* | 0.525 | 0.263 | 0.967 | 0.688 | 0.205 | 0.197 | 0.273 | 0.208 |  |
| *Maternal Education at Birth* | 0.512 | 0.087 | 0.434 | 0.142 | 0.061 | 0.801 | 0.604 | 0.986 |  |
| *Maternal Smoking* | 0.235 | 0.317 | **0.011** | 0.147 | 0.705 | 0.143 | 0.094 | 0.196 |  |
| *Gest. Age at Sampling* | 0.094 | 0.067 | 0.356 | 0.722 | 0.998 | **0.011** | 0.336 | 0.611 |  |
| *Sampling Quarter* | 0.078 | 0.094 | 0.933 | 0.829 | 0.997 | 0.162 | 0.097 | 0.770 |  |

Abbreviations: **Psych**: Psychiatric; **Hist**; History; **BMI**: Body Mass Index; **A2M**: α-2 macroglobulin; **CRP**: C-reactive protein; **FER**: ferritin; **FIB**: fibrinogen; **PCT**: procalcitonin; **SAA**: serum amyloid A; **SAP**: serum amyloid P; and **tPA**: tissue plasminogen activator.

**Supplementary Table 6.** P-values for non-linearity in restricted cubic spline analyses. Not all relationships we considered are necessarily non-linear. To assess evidence for non-linear relationships, we tested the null-hypothesis that all spline terms that would indicate a change in the slope of the relationship (i.e., all but the first spline term) are equal to zero using a Wald test.

|  | *Any ASD* | | *ASD only* | | *ASD w/ ID* | | *ASD w/ ADHD* | |
| --- | --- | --- | --- | --- | --- | --- | --- | --- |
|  | unadjusted | adjusted | unadjusted | adjusted | unadjusted | adjusted | unadjusted | adjusted |
| *A2M* | 0.099 | 0.198 | **0.028** | 0.082 | 0.867 | 0.827 | 0.233 | 0.298 |
| *CRP* | 0.083 | 0.298 | 0.291 | 0.606 | 0.905 | 0.442 | **0.034** | **0.038** |
| *FER* | 0.09 | 0.055 | 0.97 | 0.695 | 0.084 | **0.036** | 0.133 | 0.111 |
| *FIB* | 0.651 | 0.807 | 0.929 | 0.807 | 0.699 | 0.889 | 0.566 | 0.854 |
| *PCT* | 0.871 | 0.824 | 0.492 | 0.64 | 0.691 | 0.491 | 0.902 | 0.915 |
| *SAA* | 0.298 | 0.307 | 0.717 | 0.552 | 0.197 | 0.252 | 0.615 | 0.526 |
| *SAP* | 0.208 | 0.314 | 0.343 | 0.691 | 0.415 | 0.502 | **0.034** | **0.038** |
| *tPA* | 0.99 | 0.945 | 0.402 | 0.301 | 0.584 | 0.529 | 0.921 | 0.638 |

Abbreviations: **ASD**: autism spectrum disorders; **ADHD**: attention-deficit/hyperactivity disorder; **ID**: intellectual disability**; A2M**: α-2 macroglobulin; **CRP**: C-reactive protein; **FER**: ferritin; **FIB**: fibrinogen; **PCT**: procalcitonin; **SAA**: serum amyloid A; **SAP**: serum amyloid P; and **tPA**: tissue

plasminogen activator.
